# Desloratadine Rescues Schizophrenia-like Phenotypes by Inhibiting the Pathogenic 5-HT2AR-PI3K/AKT/mTOR Signaling Axis

**DOI:** 10.1007/s12035-026-06027-z

**Published:** 2026-06-24

**Authors:** Yuhan Yao, Weiliang Zhao, Junyang Chen, Shentong Wang, Zhiyao Song, Lin Sun, Yubo Hu, Longyun Li

**Affiliations:** Department of Anesthesiology, China-Japan Union Hospital of Jilin University, Changchun, 130033 Jilin People’s Republic of China

**Keywords:** 5-HT2AR, Schizophrenia, Desloratadine, Single-nucleus RNA sequencing, PI3K/AKT/mTOR pathway

## Abstract

The 5-HT2A receptor (5-HT2AR) is a validated target in schizophrenia (SCZ); however, the therapeutic potential of repurposed drugs targeting this pathway remains underexplored. Here, we show that the antihistamine desloratadine (DLT) robustly ameliorates a full spectrum of SCZ-like behavioral deficits in an MK-801-induced male mouse model. Single-nucleus RNA sequencing (snRNA-seq) analysis revealed 5-HT2AR expression specifically within neuronal populations of the medial prefrontal cortex (mPFC). Mechanistically, these behavioral impairments were associated with a specific upregulation of 5-HT2AR in mPFC neurons—a molecular pathology reversed by DLT. Targeted overexpression of 5-HT2AR in mPFC neurons was sufficient to recapitulate the SCZ-like phenotypes, which are rescued by DLT, establishing a causal role for this receptor in disease pathology. Through integrated transcriptomic and biochemical analyses, we identified the PI3K/AKT/mTOR pathway as a key downstream effector of 5-HT2AR. We demonstrated that 5-HT2AR-mediated activation of this pathway drives neuroinflammation, apoptosis, and long-term potentiation impairments—effects that were effectively blocked by DLT. In a definitive pharmacological reversal experiment, activation of AKT with SC79 completely abrogated DLT’s therapeutic efficacy, both behaviorally and molecularly. Collectively, our findings reveal that DLT exerts its therapeutic effects by suppressing a pathogenic feed-forward loop, wherein 5-HT2AR activates the PI3K/AKT/mTOR pathway, which may, in turn, sustain its own aberrant expression. This study provides a compelling rationale for repurposing desloratadine for SCZ and validates the 5-HT2AR–PI3K/AKT/mTOR signaling axis as a pivotal, druggable target for therapeutic intervention.

**Graphical Abstract:**

mPFC neuronal 5-HT2AR upregulation activates PI3K/AKT/mTOR signaling to drive neuroinflammation, apoptotic stress, synaptic dysfunction, and schizophrenia-like behaviors. Desloratadine attenuates these phenotypes by suppressing the pathway, and AKT activation counteracts the rescue (created using BioRender).

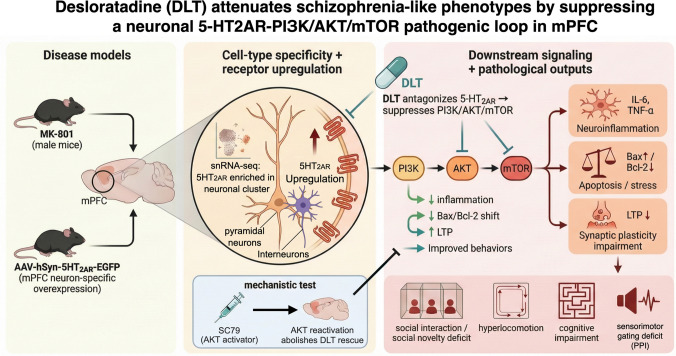

**Supplementary Information:**

The online version contains supplementary material available at 10.1007/s12035-026-06027-z.

## Introduction

Schizophrenia (SCZ) is a severe and complex psychiatric disorder characterized by a constellation of positive, negative, and cognitive symptoms that collectively result in profound functional disability [1, 2]. For decades, therapeutic strategies have primarily targeted dopamine D2 receptors [3–5]. While this approach is effective for many positive symptoms, it has proven largely inadequate for treating the debilitating negative and cognitive deficits that primarily drive poor long-term outcomes [6–8]. Furthermore, existing antipsychotics are burdened by significant side effects, including metabolic dysregulation and severe motor impairments, highlighting the urgent need for novel therapeutics that engage non-dopaminergic targets and offer broader efficacy with improved safety profiles.

Among alternative targets, the serotonin 5-HT2A receptor (5-HT2AR) has emerged as a focal point in modern SCZ research [9–11]. The involvement of this receptor in psychosis is strongly supported by pharmacological evidence; hallucinogens such as LSD are potent 5-HT2AR agonists, while the superior clinical profiles of many atypical antipsychotics are attributed, at least in part, to their strong 5-HT2AR antagonism [12, 13]. This validates 5-HT2AR as a therapeutically relevant target. Nevertheless, a critical knowledge gap remains. The precise intracellular signaling cascades dysregulated downstream of 5-HT2AR that contribute to neuronal dysfunction and the manifestation of SCZ symptoms remain incompletely understood. A deeper mechanistic understanding of this axis is essential for the rational development of next-generation therapies that more precisely target disease pathophysiology.

Drug repurposing offers an efficient strategy to address this therapeutic need. Desloratadine (DLT), a widely used second-generation antihistamine with a well-established safety profile, was recently shown to possess off-target activity as a selective 5-HT2AR antagonist [14, 15]. While DLT is primarily known for its H1 receptor (H1R) antagonism, this unexpected property has been associated with neuroprotective and anti-inflammatory effects in preclinical models of other neurodegenerative disorders, such as Alzheimer’s disease, where 5-HT2AR dysregulation is also implicated [16]. These findings provide a compelling rationale to investigate DLT in SCZ, a disease that shares overlapping pathophysiological features. To date, however, the therapeutic potential of DLT in SCZ has not been explored. It remains an open and important question whether the 5-HT2AR antagonistic properties of DLT can be leveraged to treat SCZ-related behavioral and cellular abnormalities.

To establish a mechanistic link between receptor-level dysfunction and disease phenotype, it is critical to identify the key intermediary signaling pathways. The phosphatidylinositol 3-kinase (PI3K)/AKT/mTOR pathway is a central regulator of cellular metabolism, survival, synaptic plasticity, and inflammation—all of which are profoundly disrupted in SCZ [17–19]. Although dysregulation of the PI3K/AKT/mTOR pathway has been previously reported in SCZ [20, 21], a direct causal connection between aberrant 5-HT2AR signaling and activation of this pathway in the context of SCZ has not been definitively established [22, 23]. Clarifying whether this signaling hub functions as a primary downstream effector of 5-HT2AR-mediated pathology would both illuminate disease mechanisms and identify a highly specific, druggable target for therapeutic intervention.

Here, we systematically investigated the therapeutic potential and underlying mechanisms of action of DLT in SCZ. We demonstrated that DLT robustly reversed a broad spectrum of behavioral abnormalities in a pharmacological mouse model of SCZ. Mechanistically, snRNA-seq analysis revealed 5-HT2AR expression specifically within neuronal populations of the medial prefrontal cortex (mPFC) and we identified a specific upregulation of 5-HT2AR in mPFC neurons as a core molecular pathology. Using virally mediated overexpression, we provide causal evidence that elevated 5-HT2AR levels in mPFC neurons are sufficient to drive SCZ-like behaviors. Critically, we found that 5-HT2AR exerts its pathogenic effects through potent activation of the PI3K/AKT/mTOR pathway, which in turn promotes neuroinflammation, apoptosis, and impairments in synaptic plasticity—a pathological cascade strongly attenuated by DLT. In a mechanistically informative reversal experiment, we showed that pharmacological activation of AKT partially reversed the therapeutic actions of DLT. Our findings identify a pathogenic feed-forward signaling axis in this SCZ-like model and provide preclinical support for desloratadine as a repurposing candidate that may help disrupt this pathological loop.

## Materials and Methods

### Animals

All experiments were conducted using male C57BL/6 J mice (8–10 weeks old at the start of experiments) obtained from Center for Experimental Animals, Jilin University. Animals were housed in a specific pathogen-free facility on a 12-h light/dark cycle with ad libitum access to standard chow and water. All animal procedures were performed in strict accordance with the guidelines of the National Institutes of Health Guide for the Care and Use of Laboratory Animals and were approved by the Institutional Animal Care and Use Committee of Jilin University.

### Animal Models and Drug Administration

To induce SCZ-like phenotypes, mice were randomly assigned to experimental groups. The Schizophrenia (SCZ) model was established by daily intraperitoneal (i.p.) injections of MK-801 (2 mg/kg/day; MedChemExpress) for 6 consecutive days (Days 1–6). Pharmacological treatment with Desloratadine (DLT) or vehicle commenced after the completion of MK-801 administration (starting on Day 14) and continued for 2 weeks (Days 14–28). DLT (20 mg/kg; MedChemExpress) was suspended in a vehicle of 10% DMSO with 90% physiological saline and administered by oral gavage.

The study comprised three distinct experimental groups: Control (Con) Group: Received daily saline injections (i.p.) followed by daily vehicle gavage (10% DMSO). SCZ Group: Received daily MK-801 injections (i.p.) followed by daily vehicle gavage (10% DMSO). SCZ + DLT Group: Received daily MK-801 injections (i.p.) followed by daily DLT gavage (20 mg/kg). Behavioral testing for all groups commenced after the completion of the 2-week treatment period.

### Stereotactic Surgery

Mice were deeply anesthetized with isoflurane (2% for induction, 1.5% for maintenance) and secured in a stereotaxic frame (RWD Life Science). For virally-mediated overexpression or pharmacological manipulation, a small craniotomy was performed over the mPFC. All adeno-associated viruses (AAVs), including high-titer AAV-hSyn-5HT2AR-EGFP and the corresponding empty vector control, were custom-packaged by BrainVTA. AAVs (200 nL per hemisphere) or SC79 (100 µM in 0.2 µL aCSF, MedChemExpress, HY-18749) were infused bilaterally into the mPFC (AP: + 1.9 mm; ML: ± 0.4 mm; DV: −2.5 mm, relative to bregma) using a 33-gauge needle connected to a microinfusion pump (100 nL/min). The needle was left in situ for 10 min post-infusion to allow for diffusion before being slowly withdrawn. For the pharmacological reversal experiments, SC79 was administered as an acute bilateral intra-mPFC infusion 30 min before the corresponding behavioral session or tissue collection. Specifically, for non-MWM behavioral tests, SC79 was infused 30 min before each behavioral test. For the Morris Water Maze reversal experiment, SC79 was infused 30 min before each daily acquisition session and again 30 min before the probe trial. Tissue samples were collected after completion of the behavioral assessments.

### Behavioral Analyses

All behavioral assessments were conducted during the light cycle by an experimenter blinded to the experimental groups. Arenas were thoroughly cleaned with 70% ethanol between trials. To minimize the confounding effects of stress from prior tasks, the same cohort of mice underwent the behavioral battery in a specific sequence, arranged from the least stressful to the most stressful: Open Field Test, Three-Chamber Social Interaction Test, Grooming Test, Prepulse Inhibition (PPI) Test, and finally the Morris Water Maze (MWM). A strict inter-test interval of 24 h was maintained between each distinct behavioral assay to allow for adequate rest and prevent carry-over fatigue.

#### Open Field Test (OFT)

General locomotor activity and anxiety-like behavior were assessed by placing mice into a 41 × 41 × 41 cm arena for 10 min. An automated video-tracking system (SMART 3.0, Panlab) quantified total distance traveled and time spent in the center (20 × 20 cm) versus the periphery.

#### Three-Chamber Social Interaction Test

Sociability and social novelty preference were assessed in a standard three-chambered apparatus. Time spent exploring a novel mouse (Stranger 1) versus an empty enclosure (sociability) and a second novel mouse (Stranger 2) versus the now-familiar Stranger 1 (social novelty) was quantified. The social index was calculated as follows: for Sociability (Session 1), (Time in Stranger 1 zone—Time in Empty zone)/(Total interaction time); for Social Novelty (Session 2), (Time in Stranger 2 zone—Time in Stranger 1 zone)/(Total interaction time).

#### Grooming Test

Stereotypy was assessed by placing mice individually into a clean cage and manually scoring the cumulative time spent engaged in grooming behavior over a 10-min observation period.

#### Prepulse Inhibition (PPI) Test

Sensorimotor gating was assessed using a startle response system. Mice were placed in a restrainer within a sound-attenuating chamber. After a 5-min acclimatization period (65 dB background noise), mice were subjected to a series of trials: (1) Pulse alone (120 dB, 40 ms); (2) Prepulse + Pulse (76, 79, or 85 dB prepulse for 20 ms, followed 100 ms later by the 120 dB pulse); and (3) No stimulus. Trials were presented in a pseudorandom order. %PPI was calculated as: [1—(Startle amplitude_prepulse_/Startle amplitude_pulse alone_)] × 100.

#### Morris Water Maze (MWM)

Spatial learning and memory were assessed in a 120 cm diameter pool. The acquisition phase consisted of 5 consecutive days of training (4 trials/day) to locate a hidden platform. On day 6, a 60 s probe trial was conducted with the platform removed. Escape latency, platform crossings, and percentage of time in the target quadrant were analyzed.

### Single-nucleus RNA Sequencing (snRNA-seq) and Bioinformatic Analysis

Freshly dissected mPFC tissue was flash-frozen. Nuclei were isolated using a Dounce homogenizer in ice-cold EZ Lysis Buffer (Sigma-Aldrich), followed by density gradient centrifugation. Single-nucleus libraries were generated using the Chromium Next GEM Single Cell 3' Reagent Kits v3.1 (10 × Genomics) and sequenced on an Illumina NovaSeq 6000 platform. Raw reads were aligned and quantified using Cell Ranger. Raw gene expression matrices were processed using the Seurat R package (v4.0). Quality control was rigorously applied: nuclei with fewer than 200 or more than 5000 detected genes, or with > 5% mitochondrial counts, were excluded. Batch correction was performed using the Harmony algorithm to integrate datasets from different biological replicates. Dimensionality reduction was conducted using Principal Component Analysis (PCA) on the top 2,000 variable features. The top 20 principal components were selected for clustering (Louvain algorithm, resolution 0.3–0.5) and UMAP visualization. Cell types were annotated based on canonical markers: *Slc17a7* (Excitatory neurons), *Gad1/Gad2* (Inhibitory neurons), *Aqp4* (Astrocytes), *Cx3cr1* (Microglia), *Mbp* (Oligodendrocytes), and *Pdgfra* (OPCs). For neuronal subclustering, the neuronal cluster was extracted and re-clustered to distinguish Excitatory and Inhibitory lineages. Pathway scores were calculated using the AddModuleScore function based on PI3K/AKT/mTOR pathway genes gene sets.

For snRNA-seq, nuclei from three mice per group were pooled prior to library construction, resulting in one library per condition. Differential expression was computed at the nucleus level using Seurat (Wilcoxon rank-sum) to generate cell-type–resolved candidate gene lists. Given the pooled design, animal-level pseudo-bulk modeling is not feasible; thus, we interpret snRNA-seq results as exploratory/discovery and rely on independent animal-level validation assays (WB/ELISA/IF/qPCR) for statistical inference.

### Biochemical and Histological Analyses

#### Western Blotting

mPFC and hippocampus tissues were homogenized in ice-cold RIPA buffer supplemented with protease and phosphatase inhibitor cocktails (Roche). Protein concentrations were quantified using a BCA assay (Beyotime). Equal amounts of protein (20 µg) were resolved by SDS-PAGE, transferred to PVDF membranes, and blocked in 5% non-fat milk. Membranes were incubated with primary antibodies overnight at 4 °C, followed by HRP-conjugated secondary antibodies for 1 h at room temperature. Chemiluminescent signal was detected using Tanon 4600. Band densitometry was quantified using ImageJ software. 5-HT2AR (1:1000, proteintech, #67015), MAP2 (1:1000, proteintech, #26438), BDNF (1:2000, proteintech, #28205), cFos (1:1000, proteintech, #66590), PSD95 (1:1000, proteintech, #30255), Phospho-PI3 Kinase (1:1000, CST, #17366), PI3 Kinase (1:1000, CST, #4257), Phospho-AKT (1:1000, proteintech, #66444), AKT (1:5000, proteintech, #60203), Phospho-mTOR (1:1000, proteintech, #67778), mTOR (1:5000, proteintech, #28273), GAPDH (1:5000, proteintech, #60004). Micro-punches of the mPFC were collected. Due to the limited amount of protein extracted from a single mouse mPFC, tissues from 3–4 mice within the same experimental group were pooled to form one biological replicate. A total of 3 independent biological pools were analyzed per group.

#### ELISA

Protein levels of TNF-a (ABclonal, RM17709) and IL-6 (ABclonal, RM17709) in mPFC lysates were quantified using ELISA kits following the manufacturer’s instructions.

#### Immunofluorescence (IF) and Microscopy

Mice were transcardially perfused with ice-cold PBS followed by 4% PFA. Brains were post-fixed, cryoprotected in 30% sucrose, and cryosectioned at 30 µm. Free-floating sections were permeabilized, blocked, and incubated with primary antibodies followed by Alexa Fluor-conjugated secondary antibodies (Invitrogen) and nuclei were counterstained with DAPI. 5-HT2AR (1:200, proteintech, #67,015), MAP2 (1:100, proteintech, #26,438). Images were acquired using a confocal microscope (FV3000, Olympus).

#### Transmission Electron Microscopy (TEM)

Small mPFC blocks were fixed in 2.5% glutaraldehyde, post-fixed in 1% osmium tetroxide, dehydrated through an ethanol series, and embedded in Epon resin. Ultrathin Sects. (70 nm) were stained with uranyl acetate and lead citrate and imaged under a Hitachi H-7650 transmission electron microscope (Tokyo, Japan).

### Electrophysiology

Acute coronal mPFC slices (300 µm) were prepared in ice-cold, oxygenated aCSF. Slices were allowed to recover for at least 1 h before recording. Field excitatory postsynaptic potentials (fEPSPs) were recorded in layer V of the prelimbic cortex. After establishing a stable 20-min baseline, long-term potentiation (LTP) was induced by a high-frequency stimulation (HFS) protocol consisting of four 100 Hz trains of 1 s duration, delivered with an inter-train interval of 10 s. The fEPSP slope was recorded for at least 60 min post-HFS. For analysis, all fEPSP slope values were normalized to the average slope of the 10-min baseline period. Data were acquired and analyzed using pCLAMP software.

### Statistical Analysis

All data are presented as mean ± SEM. Statistical analyses were performed using GraphPad Prism 10.5.0. Comparisons between two groups were conducted using a two-tailed, unpaired Student’s t-test. Comparisons among three or more groups were performed using one-way or two-way ANOVA, followed by Tukey’s or Dunnett’s multiple comparisons test as appropriate for the experimental design. A P-value less than 0.05 was considered statistically significant. Levels of significance are denoted as *P < 0.05, **P < 0.01, and ***P < 0.001.

## Results

### DLT Ameliorates Behavioral Deficits in the MK-801-induced Model of Schizophrenia

**Fig. 1:**
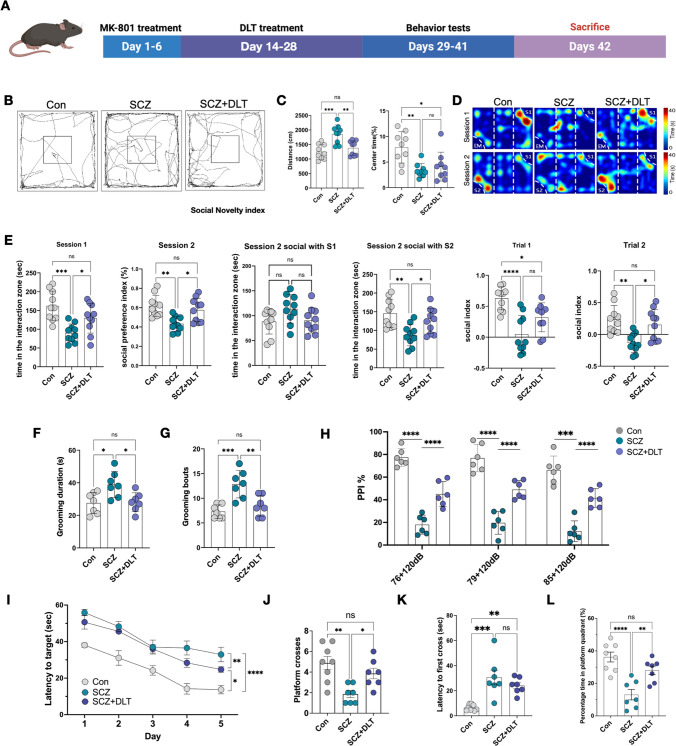
DLT Ameliorates a broad range of schizophrenia-like behavioral deficits in the MK-801 mouse model. (**A**) Schematic of the experimental design for the MK-801-induced schizophrenia (SCZ) model and desloratadine (DLT) treatment timeline. (**B**) Representative locomotor track plots from the open field test for control (Con), SCZ, and DLT-treated SCZ mice. (**C**) Quantification of total distance traveled, and percentage of time spent in the center zone during the open field test. (**D**) Representative heatmaps during Session 1 (sociability) and Session 2 (social novelty), illustrating interaction time in different zones of the apparatus. (**E**) Quantification of social behavior from the three-chamber test, including time spent in the interaction zone during the Session 1 (sociability) and Session 2 (social novelty), and interaction time. Grooming test for stereotypy, showing (**F**) total time and (**G**) frequency of grooming events. (**H**) PPI test of acoustic startle at different prepulse intensities (76, 79, and 85 dB) with a 120 dB startle pulse. (**I**) Escape latency over the 5-d training phase, showing impaired learning in the SCZ group that is improved by DLT. Quantification of (**J**) platform crosses, (**K**) latency to first cross the platform location, and (**L**) percentage of time spent in the target quadrant during the probe trial. All data are presented as mean ± SEM (n = 7–10 mice per group). Statistical significance was assessed using two-way ANOVA with repeated measures (for MWM training) or one-way ANOVA followed by Tukey’s post-hoc test (for all other panels). *P < 0.05, **P < 0.01, ***P < 0.001, ****P < 0.0001. ns, not significant

To evaluate the therapeutic potential of DLT, we first established a pharmacological mouse model of SCZ using MK-801, followed by DLT administration according to the experimental timeline (Fig. 1A). In the open-field test, MK-801 markedly increased total distance traveled, and DLT significantly attenuated this hyperlocomotion toward control levels; however, the reduction in center-zone time was not fully restored (Fig. 1B–C). In the three-chamber social interaction test, MK-801–treated mice exhibited impaired sociability (Session 1) and reduced social novelty (Session 2), as reflected by decreased interaction time and lower sociability/social novelty indices calculated from interaction time; DLT significantly improved these social deficits (Fig. 1D–E). MK-801 also increased repetitive self-grooming, including grooming duration and grooming bouts, both of which were reduced by DLT (Fig. 1F–G). Consistent with sensorimotor-gating impairment, MK-801 robustly decreased PPI across prepulse intensities, and DLT significantly improved PPI (Fig. 1H). In the MWM test, MK-801–treated mice showed impaired spatial learning during acquisition and reduced spatial memory in the probe trial; DLT improved learning performance and probe measures (platform crossings and time spent in the target quadrant), with no improvement in latency to the first platform crossing (Fig. 1I–L). Consistency with these quantitative metrics, qualitative analysis of swim paths revealed that SCZ mice exhibited disorganized search trajectories, which were notably improved by DLT treatment (Fig. S1).

### DLT Reverses 5-HT2AR Upregulation and Associated Synaptic Pathology in the SCZ Model

**Fig. 2:**
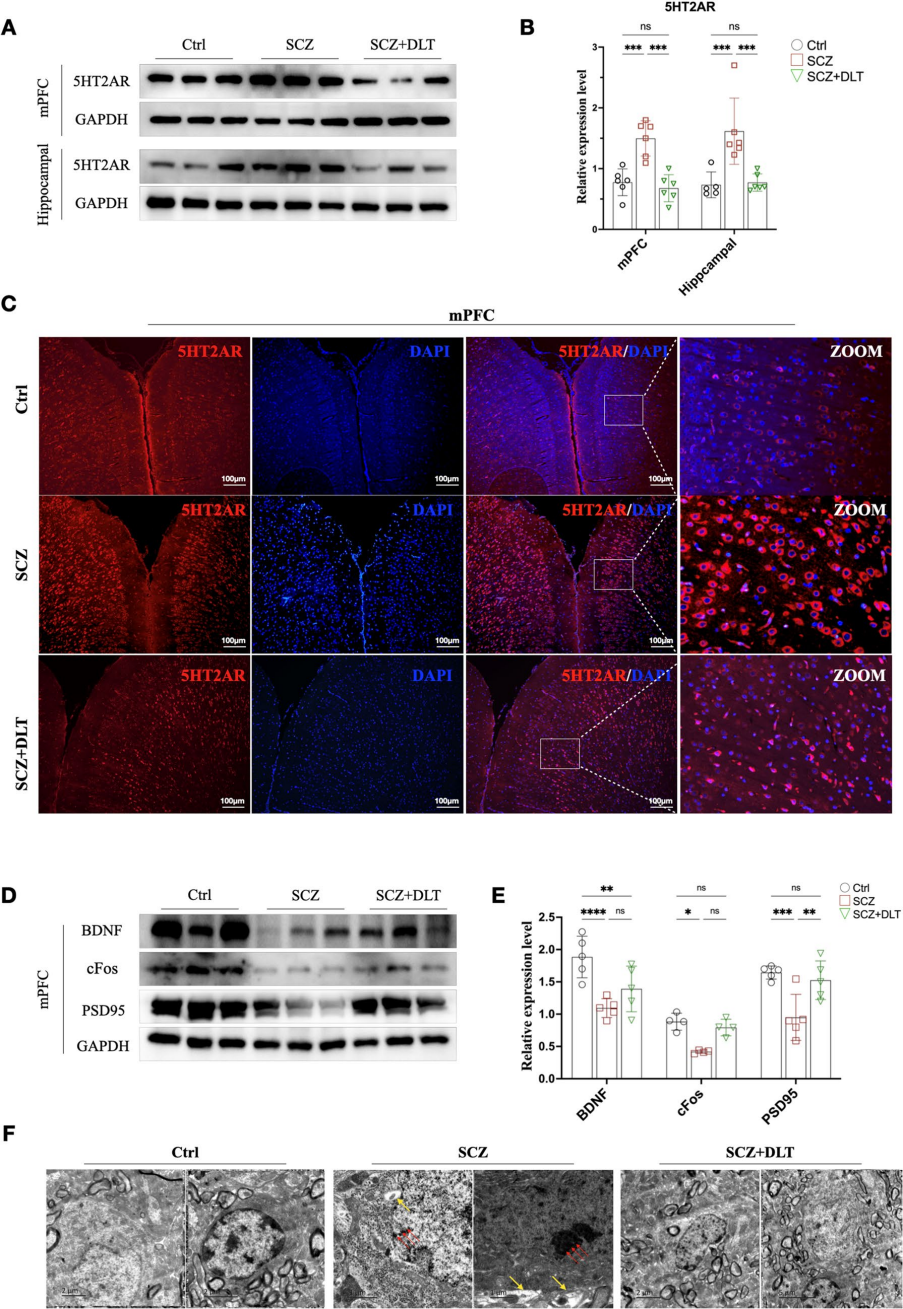
DLT rescues 5-HT2AR upregulation and associated synaptic pathology in the SCZ model. (**A**) Representative Western blots showing the protein levels of 5-HT2AR in the medial prefrontal cortex (mPFC) and hippocampus of control (Ctrl), schizophrenia model (SCZ), and DLT-treated SCZ mice (SCZ + DLT). (**B**) Densitometric quantification of 5-HT2AR protein levels, normalized to GAPDH. (**C**) Representative immunofluorescence images showing 5-HT2AR expression (red) in the mPFC across the control (Ctrl), schizophrenia model (SCZ), and DLT-treated (SCZ + DLT) groups. Nuclei are counterstained with DAPI (blue). Scale bars, 100 µm (main) and 50 µm (zoom). (**D**) Representative Western blots showing protein levels of the neurotrophic factor BDNF, the neuronal activity marker c-Fos, and the postsynaptic protein PSD95 in the mPFC from control, schizophrenia model, and DLT-treated SCZ mice. (**E**) Quantification of the levels of BDNF, c-Fos, and PSD95 from control, schizophrenia model, and DLT-treated SCZ mice. (**F**) Representative TEM images showing the ultrastructural changes in mPFC neurons. Left (Ctrl): Neurons exhibit normal morphology with round, euchromatic nuclei and intact cytoplasm (Scale bar = 2 µm). Middle (SCZ): SCZ mice display distinctive signs of neuronal degeneration. The high-magnification panel (left) reveals chromatin condensation (red arrows) aggregated near the nuclear envelope and cytoplasmic vacuolation (yellow arrows). The right panel shows the disorganized somatic context (Scale bar = 1 µm). Right (SCZ + DLT): DLT treatment markedly reduces these degenerative features, restoring nuclear integrity and reducing vacuolation (Scale bars = 2 µm and 5 µm). All data are presented as mean ± SEM (n = 5 mice per group). Statistical significance was assessed using a one-way ANOVA followed by Tukey’s post-hoc test. *P < 0.05, **P < 0.01, ***P < 0.001, ****P < 0.0001. ns, not significant

To investigate the molecular underpinnings of the behavioral deficits, we assessed 5-HT2A receptor (5-HT2AR) expression in the medial prefrontal cortex (mPFC) and hippocampus. Western blot analysis revealed significant upregulation of 5-HT2AR protein in both brain regions of SCZ mice relative to controls; importantly, DLT treatment completely normalized this aberrant expression (Fig. 2A, B). Immunofluorescence staining confirmed the subcellular localization of 5-HT2AR in the mPFC and hippocampus (Fig. 2C, D), and a comparative view of 5-HT2AR expression across all experimental groups is provided in Fig. S4. To determine whether this molecular pathology was associated with synaptic and neuronal damage, we next examined key markers. Western Blot analysis showed that SCZ mice exhibited significantly reduced levels of BDNF, cFos, and PSD95 compared to controls. Following DLT treatment, PSD95 levels were significantly restored (*p* < 0.01). Although BDNF levels showed an increasing trend in the DLT-treated group, this change did not reach statistical significance (*p* > 0.05; Fig. 2E, F). To directly visualize ultrastructural changes, we performed TEM on mPFC tissue. SCZ mice exhibited distinct signs of neuronal degeneration, characterized by chromatin condensation and cytoplasmic vacuolation, indicative of cellular stress (Fig. 2G). Remarkably, DLT treatment substantially reversed these pathological ultrastructural changes, restoring nuclear integrity and reducing vacuolation. These findings suggest that DLT exerts therapeutic effects by counteracting 5-HT2AR upregulation and preserving neuronal and synaptic integrity in brain circuits critical for cognition.

### Single-Nucleus Sequencing Reveals Specific Upregulation of 5-HT2AR in mPFC Neurons

**Fig. 3:**
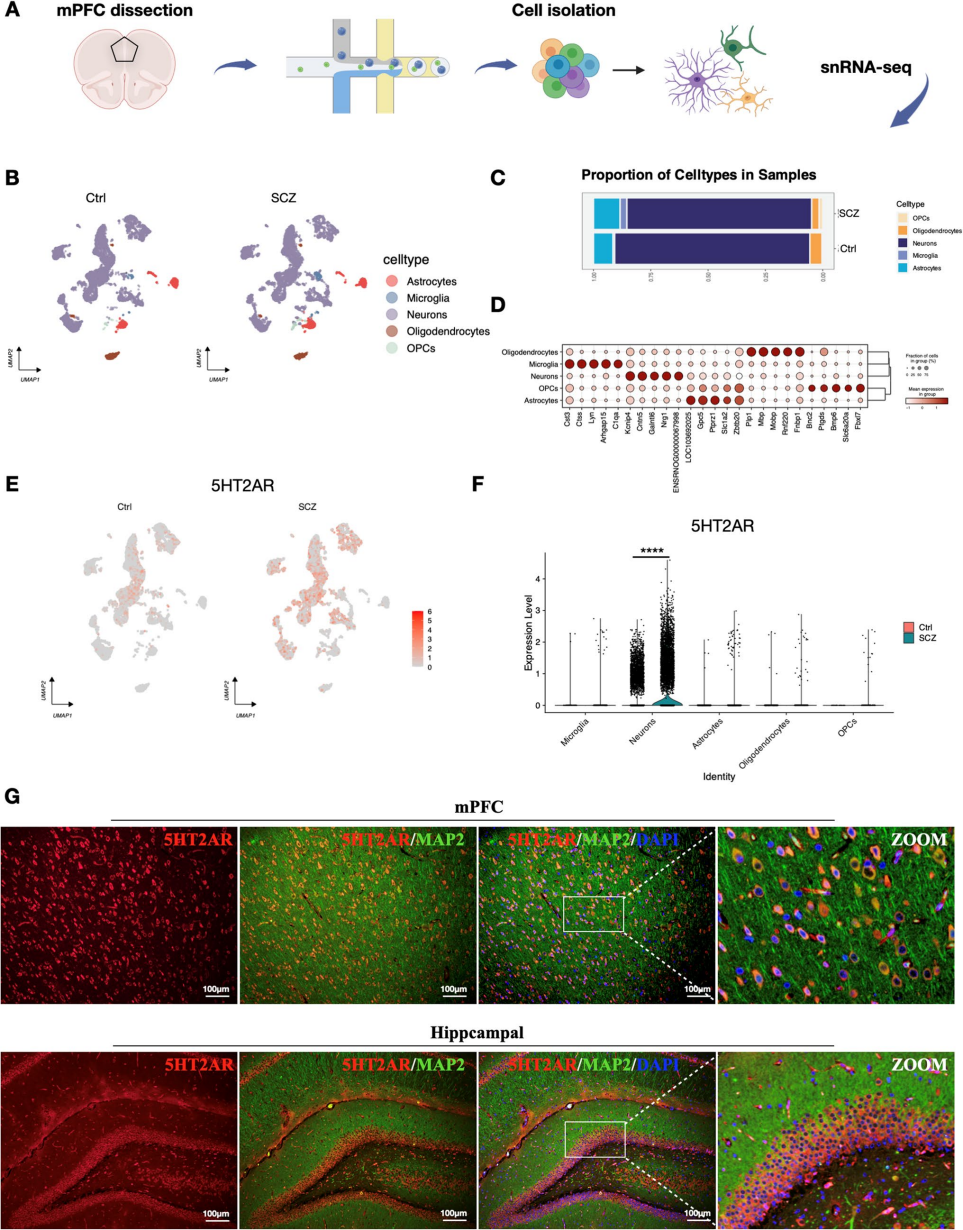
SCZ model exhibits neuron-specific 5-HT2AR upregulation in the mPFC. (**A**) Schematic overview of the snRNA-seq workflow, from microdissection of the medial prefrontal cortex (mPFC) to cell isolation and sequencing. (**B**) UMAP plots of all nuclei isolated from the mPFC of control (Ctrl) and schizophrenia model (SCZ) mice. Nuclei are colored by annotated cell type (Neurons, Astrocytes, Microglia, Oligodendrocytes, OPCs). (**C**) Bar plot showing the relative proportions of each cell type in the Ctrl and SCZ samples. (**D**) Dot plot displaying the expression of canonical marker genes used to annotate the major cell clusters. The size of the dot represents the percentage of cells in the cluster expressing the gene, and the color represents the average expression level. (**E**) Feature plots showing the expression of *Htr2a* (the gene encoding 5-HT2AR) overlaid on the UMAP plots, visually demonstrating its enrichment in the neuron cluster and its increased expression in the SCZ model. (**F**) Violin plot quantifying the normalized expression level of *Htr2a* across all identified cell types, confirming a significant and specific upregulation within the neuronal population of SCZ mice. (**G**) Dual-label immunofluorescence staining in the mPFC and hippocampus from the SCZ group to validate the localization of 5-HT2AR protein (red) on neurons, marked by MAP2 (green). Nuclei are counterstained with DAPI (blue). High-magnification insets (Zoom) show extensive colocalization on the soma and proximal dendrites of neurons. Scale bars, 50 µm (main images) and 20 µm (zoom insets). For snRNA-seq, data from three mice per group were pooled. For immunofluorescence, images are representative of four mice per group. Statistical significance (F) was determined using the Wilcoxon rank-sum test. ****P < 0.0001

To complement our biochemical findings with transcriptomic cell-type resolution and dissect the cellular basis of the molecular alterations observed in the mPFC, we performed single-nucleus RNA sequencing (snRNA-seq) on tissue from both control (Ctrl) and SCZ model mice (Fig. 3A). Unbiased clustering analysis identified all major neural cell populations based on canonical marker gene expression, including neurons, astrocytes, microglia, oligodendrocytes, and OPCs (Fig. 3B, D). While the overall distribution of major neural cell populations was largely maintained, we observed a trend toward an increased proportion of astrocytes in the SCZ model (Fig. 3C), which is consistent with the elevated neuroinflammatory state identified in our biochemical assays. Critically, we interrogated the cellular source of 5-HT2AR (*Htr2a*) expression and found that it was almost exclusively confined to the neuronal cluster (Fig. 3E). Subsequent differential expression analysis confirmed that *Htr2a* transcript levels were significantly elevated specifically within neurons of SCZ mice compared to those of controls (Fig. 3F). Further sub-clustering analysis of the neuronal population revealed that the pathogenic upregulation of *Htr2a* in the SCZ model was not restricted to a single subtype, but was significantly increased in both excitatory (*Slc17a7* +, avg_log2FC = 1.86, *p* < 0.0001) and inhibitory (Gad1/Gad2 +, avg_log2FC = 2.25, *p* < 0.0001) neurons (Fig. S2). To validate these transcriptomic findings at the protein level, we performed dual-label immunofluorescence for 5-HT2AR (red) and the neuronal marker MAP2 (green). In both the mPFC and hippocampus, we observed extensive co-localization, further supporting that 5-HT2AR protein is predominantly expressed on MAP2-positive neurons (Fig. 3G). These results provide strong evidence that the overall upregulation of 5-HT2AR observed in the MK-801-induced schizophrenia model is driven by its specific overexpression within neurons. To evaluate the pharmacological specificity of DLT within our dataset, we assessed the expression of the *Hrh1* across the mPFC transcriptomic profiles. Notably, *Hrh1* transcripts were largely undetectable in both neuronal and non-neuronal clusters under our sequencing depth, standing in sharp contrast to the robust expression and upregulation of *Htr2a* in these neuronal populations. While this transcriptomic pattern within our dataset strongly supports 5-HT2AR as a primary local target, we discuss the potential synergistic contribution of constitutive H1R blockade in the Discussion section.

### Specific Overexpression of 5-HT2AR in mPFC Neurons is Sufficient to Induce Schizophrenia-like Behaviors Reversed by DLT

**Fig. 4:**
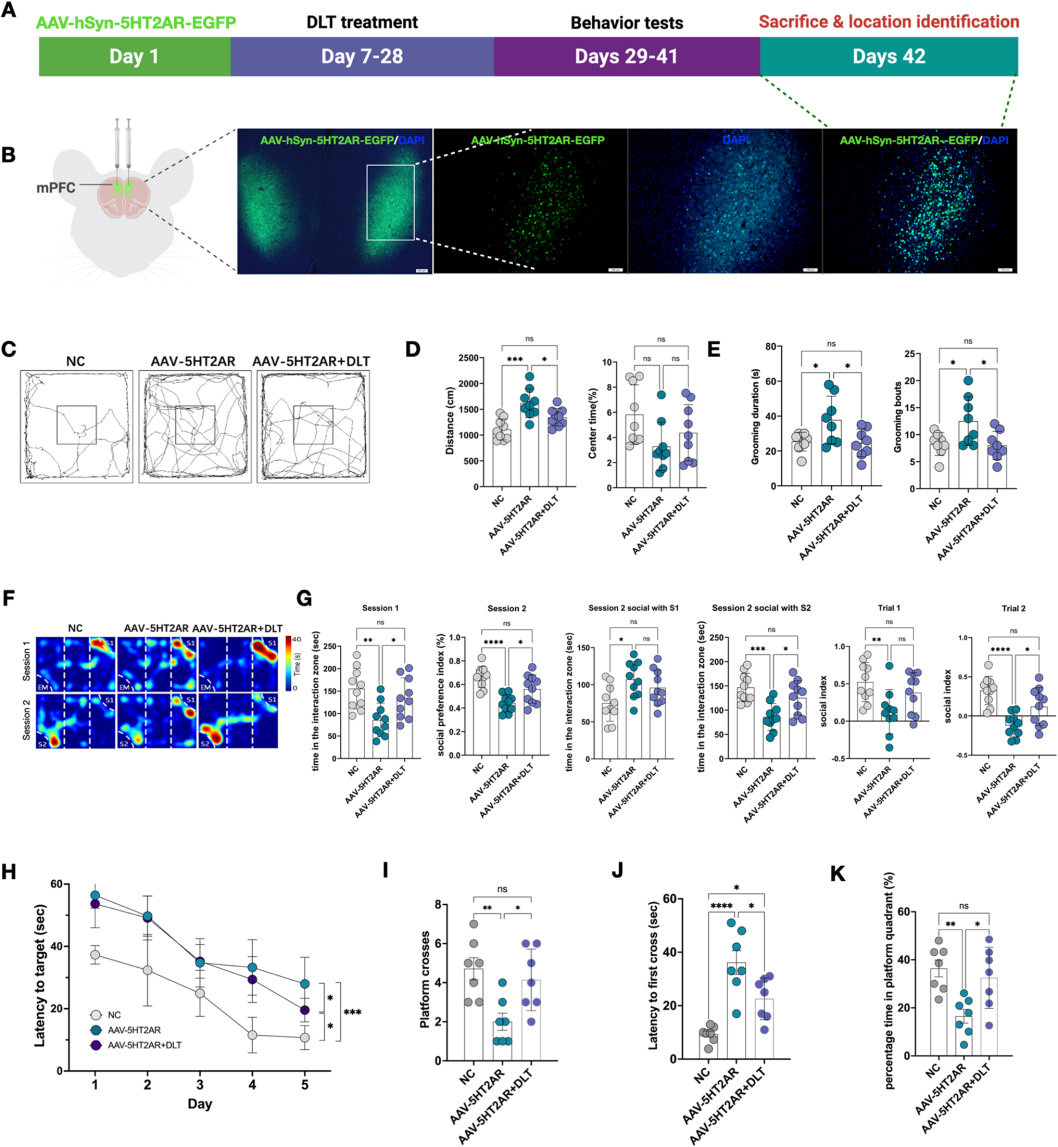
Overexpression of 5-HT2AR in mPFC neurons is sufficient to induce SCZ-like phenotypes, which are rescued by DLT. (**A**) Schematic of the experimental timeline for virally-mediated 5-HT2AR overexpression and subsequent DLT treatment. (**B**) Representative fluorescence images from the mPFC confirming successful and neuron-specific expression of the AAV-hSyn-5HT2AR-EGFP vector (green). Nuclei are counterstained with DAPI (blue). Scale bars, 200 µm (main) and 100 µm (inset). (**C-K**) Comprehensive behavioral assessment of mice with mPFC-specific 5-HT2AR overexpression (AAV-5HT2AR) compared to a negative control vector group (NC) and a DLT-treated overexpression group (AAV-5HT2AR + DLT). Open field test, showing (**C**) representative track plots and (**D**) quantification of total distance traveled and center time percentage. (**E**) Grooming test, showing quantification of grooming time and frequency. Three-chamber social interaction test, showing (**F**) representative heatmaps and (**G**) quantification of sociability and social novelty. Morris water maze test, showing (**H**) escape latency during the 5-d training phase and quantification of (**I**) platform crosses, (**J**) latency to first cross, and (**K**) percentage of time in the target quadrant during the probe trial. All data are presented as mean ± SEM (n = 7–10 mice per group). Statistical significance was assessed by one-way or two-way ANOVA with repeated measures, followed by Tukey’s post-hoc test. *P < 0.05, **P < 0.01, ***P < 0.001, ****P < 0.0001. ns, not significant. Statistical comparisons are shown relative to the NC group and between the AAV-5HT2AR and AAV-5HT2AR + DLT groups

To determine whether upregulation of 5-HT2AR in mPFC neurons is a causal factor in schizophrenia-like behaviors, we stereotactically delivered AAV-hSyn-5HT2AR-EGFP (AAV-5HT2AR) or a negative control (NC) virus into the mPFC of wild-type mice to induce neuron-specific 5-HT2AR model (Fig. 4A, B). Behavioral testing revealed that, compared to NC mice, those receiving AAV-5HT2AR exhibited the full spectrum of schizophrenia-like phenotypes. Specifically, in the open field test, AAV-5HT2AR mice showed a significant increase in total travel distance (Fig. 4C, D). In the stereotypy test, grooming time and frequency were also significantly elevated relative to those of the NC group (Fig. 4E). The three-chamber social test demonstrated that 5-HT2AR overexpression induced pronounced social deficits (Fig. 4F, G). In the MWM test, AAV-5HT2AR mice displayed impairments in spatial learning and memory (Fig. 4H–K). Importantly, systemic DLT treatment robustly rescued all of these behavioral deficits. These results indicate that overexpression of 5-HT2AR in mPFC neurons is sufficient to drive SCZ-like behaviors and that DLT can effectively antagonize the pathological consequences of 5-HT2AR overactivation.

### DLT Blocks Pathogenic Activation of the PI3K/AKT/mTOR Pathway Driven by 5-HT2AR

**Fig. 5:**
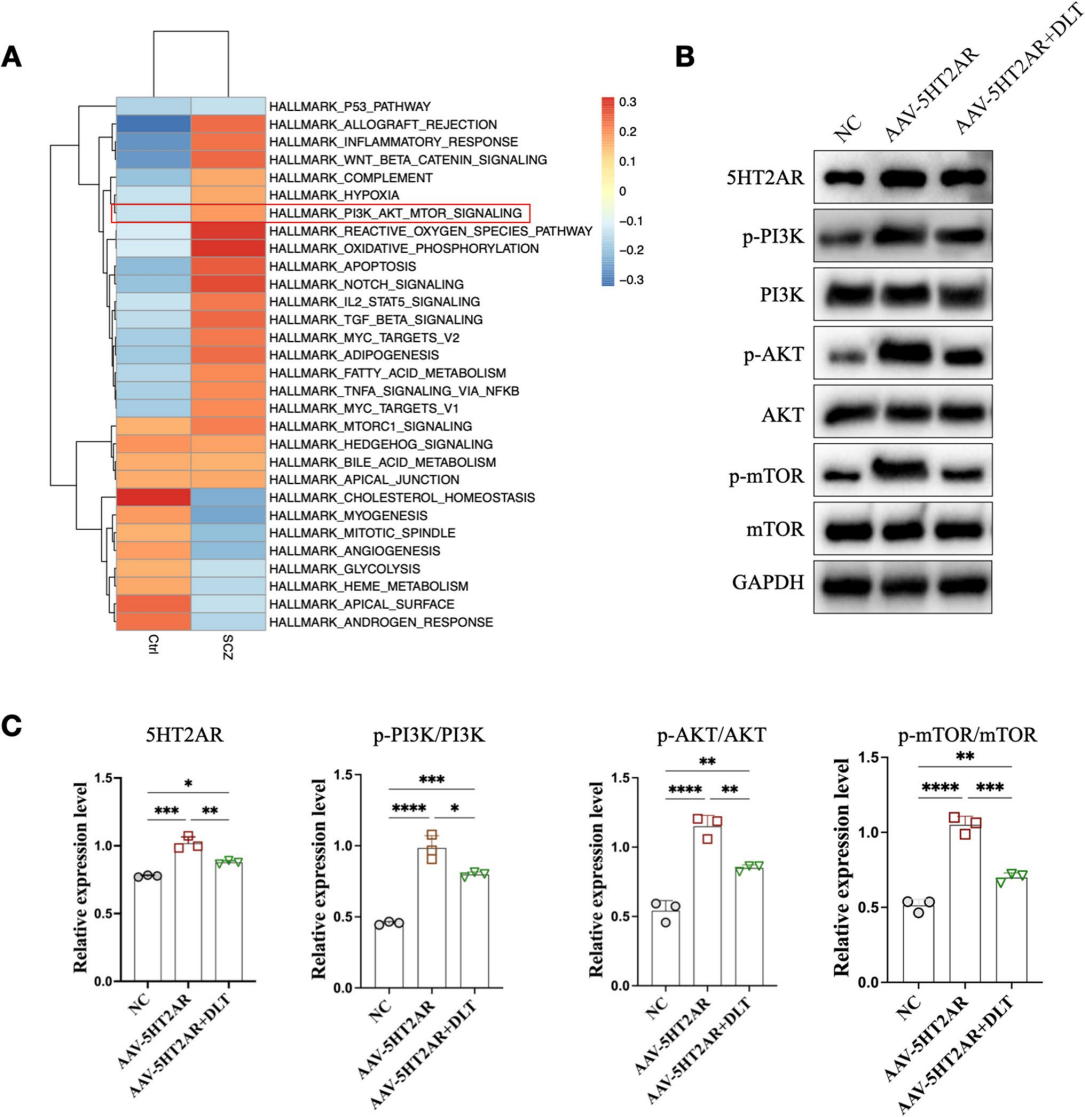
5-HT2AR drives pathogenic activation of the PI3K/AKT/mTOR pathway, a process blocked by DLT. (**A**) Heatmap of Gene Set Variation Analysis (GSVA) performed on snRNA-seq data from the medial prefrontal cortex (mPFC) of control (Ctrl) and schizophrenia model (SCZ) mice. (**B**) Representative Western blots showing the protein levels of 5-HT2AR and the phosphorylation status of key components of the PI3K/AKT/mTOR pathway in the mPFC. Lysates are from mice injected with a control vector (NC), a vector overexpressing 5-HT2AR (AAV-5HT2AR), or an overexpression vector followed by DLT treatment (AAV-5HT2AR + DLT). (**C**) Densitometric quantification of the Western blot data. Graphs show relative protein levels of 5-HT2AR and the ratios of phosphorylated to total protein for PI3K, AKT, and mTOR. Data are presented as mean ± SEM (n = 3 independent biological pools; each pool contains tissue from 3–4 mice). Statistical significance was assessed using a one-way ANOVA followed by Tukey’s post-hoc test. *P < 0.05, **P < 0.01, ***P < 0.001, ****P < 0.0001. ns, not significant

To elucidate the intracellular signaling cascade mediating the pathogenic effects of 5-HT2AR, we conducted pathway analysis of our snRNA-seq data. Gene Set Variation Analysis (GSVA) revealed significant activation of the PI3K/AKT/mTOR pathway in the mPFC of SCZ mice compared to controls (Fig. 5A). Furthermore, our snRNA-seq module scoring revealed a striking co-enrichment of *Htr2a* expression and PI3K/AKT/mTOR pathway activity specifically within the SCZ neuronal population (Fig. S3).To test whether 5-HT2AR directly activates this pathway, we examined key signaling components in our gain-of-function model. Western blot analysis showed that AAV-mediated overexpression of 5-HT2AR markedly increased phosphorylation of PI3K, AKT, and mTOR. Critically, this aberrant activation was significantly attenuated by DLT treatment (Fig. 5B, C). These findings establish a direct mechanistic link between elevated 5-HT2AR expression and activation of the PI3K/AKT/mTOR cascade, and demonstrate that DLT mediates its therapeutic effects via inhibition of this pathway.

### 5-HT2AR Overexpression Impairs Neuronal Function via Proinflammatory and Apoptotic Signaling

**Fig. 6:**
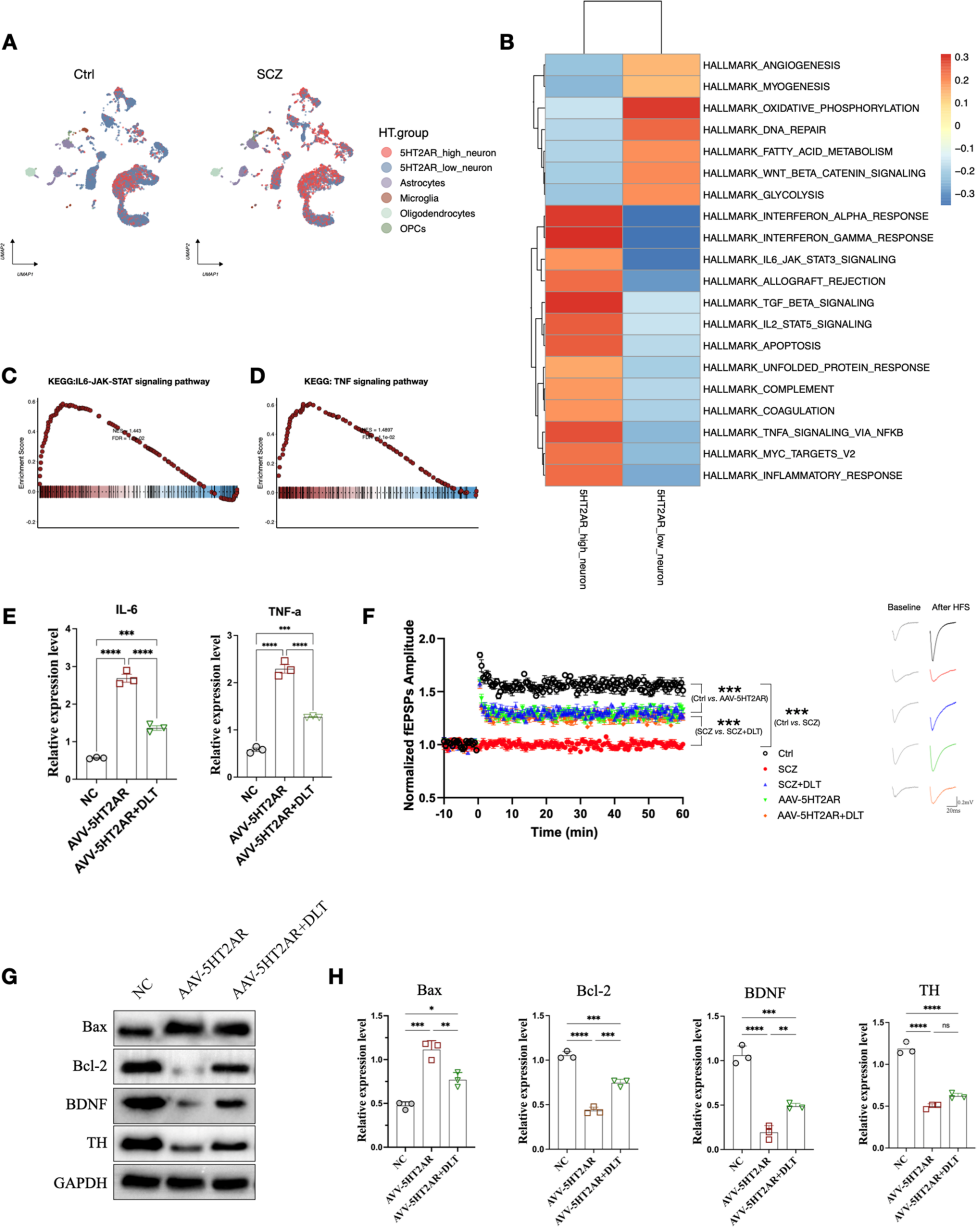
5-HT2AR overexpression impairs synaptic plasticity by promoting neuroinflammation and apoptosis. (**A**) UMAP plot derived from snRNA-seq data, illustrating the sub-clustering of neuronal populations into 5-HT2AR-high and 5-HT2AR-low expressing groups based on *Htr2a* transcript levels. (**B**) Gene Set Variation Analysis (GSVA) heatmap comparing 5-HT2AR-high and -low neuronal sub-clusters, revealing a significant enrichment of proinflammatory and apoptotic signaling pathways in the high-expressing population. Gene Set Enrichment Analysis (GSEA) plots further confirming the significant positive enrichment of (**C**) the KEGG IL-6/JAK-STAT and (**D**) the KEGG TNF-α signaling pathways in the 5-HT2AR-high neurons. (**E**) ELISA-based quantification of proinflammatory cytokines IL-6 and TNF-α in mPFC tissue lysates from the NC, AAV-mediated 5-HT2AR overexpression, and DLT treatment groups. (**F**) Summary plot of LTP recordings from acute mPFC slices. High-frequency stimulation (HFS) failed to induce LTP in slices from both the SCZ model and the 5-HT2AR overexpression (AAV-5HT2AR) model, indicating a severe synaptic plasticity deficit. DLT treatment significantly restored LTP in the SCZ model but did not improve LTP in the AAV-5HT2AR model. Representative fEPSP traces for each condition are shown to the right. (**G**) Representative Western blots showing the protein levels of the proapoptotic marker Bax, the antiapoptotic marker Bcl-2, and the neurotrophic factors BDNF and TH in the mPFC. (**H**) Quantification of the Western blot data, showing that 5-HT2AR overexpression induces a proapoptotic shift (increased Bax, decreased Bcl-2) and reduces neurotrophic support, with Bax/Bcl-2/BDNF/TH alterations being partially restored by DLT. Data are presented as mean ± SEM (n = 3–4 mice per group for molecular assays; n = 6 slices from three animals per group for electrophysiology). Statistical significance was assessed using a one-way ANOVA followed by Tukey’s post-hoc test. *P < 0.05, **P < 0.01, ***P < 0.001, ****P < 0.0001. ns, not significant

To further investigate the downstream consequences of 5-HT2AR elevation, we performed subcluster analysis of our snRNA-seq data, stratifying neurons into 5-HT2AR-high and -low populations (Fig. 6A). GSVA revealed significant enrichment of proinflammatory and apoptotic signaling pathways in 5-HT2AR-high neurons (Fig. 6B). Gene Set Enrichment Analysis confirmed strong activation of interleukin (IL)−6/JAK-STAT and tumor necrosis factor (TNF)-α signaling pathways in this population (Fig. 6C, D). These transcriptomic findings were validated at the protein level by ELISA, which demonstrated that AAV-mediated 5-HT2AR overexpression significantly increased IL-6 and TNF-α levels in the mPFC; this effect was significantly reduced by DLT (Fig. 6E). To assess functional consequences, we performed field recordings in acute mPFC slices and found that high-frequency stimulation failed to induce long-term potentiation (LTP) in slices from both SCZ and 5-HT2AR overexpression (OE) mice, indicating marked plasticity deficits. Remarkably, DLT treatment significantly restored LTP in the SCZ model. However, in the AAV-5HT2AR model, DLT failed to rescue the LTP deficits (Fig. 6F). Finally, Western blot analysis revealed that 5-HT2AR overexpression triggered a proapoptotic state, evidenced by increased Bax and decreased Bcl-2 expression, alongside reduced levels of the neuroprotective factors BDNF and TH. These molecular deficits were partially restored by DLT (Fig. 6G, H). Collectively, these findings establish that 5-HT2AR overexpression drives inflammatory and apoptotic signaling that impairs synaptic plasticity and compromises neuronal health.

### Activation of AKT Signaling Abolishes the Therapeutic and Molecular Benefits of DLT in the SCZ Model

**Fig. 7:**
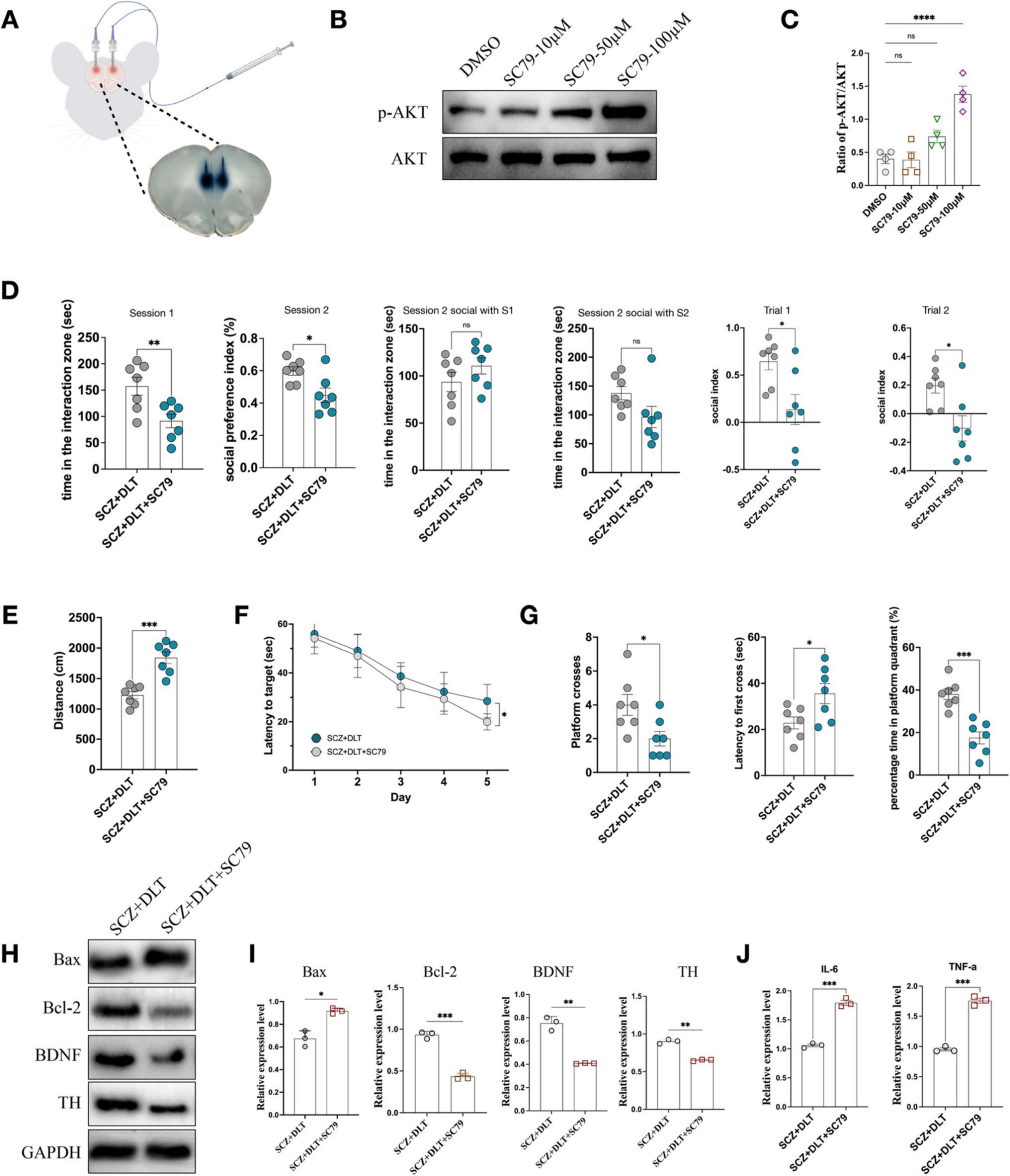
Pharmacological activation of AKT abolishes the therapeutic efficacy of DLT at both behavioral and molecular levels. (**A**) Schematic illustrating the experimental design for the pharmacological reversal study, involving stereotactic infusion of the AKT activator SC79 into the mPFC of SCZ model mice undergoing DLT treatment. (**B**) Representative Western blots and (**C**) quantification showing that intra-mPFC infusion of SC79 dose-dependently increases the phosphorylation of AKT (p-AKT) at the Ser473 site. Panels show quantification of (**D**) social behavior in the three-chamber test, (**E**) locomotor activity in the open-field test, and (**F, G**) spatial learning and memory performance in the MWM test. (**H, I**) Western blot analysis and corresponding quantification of key neuroprotective and apoptosis-related proteins in mPFC lysates. SC79 cotreatment reverses DLT's beneficial effects, increasing the proapoptotic protein Bax and decreasing the antiapoptotic protein Bcl-2, as well as the neurotrophic factors BDNF and TH. (**J**) ELISA quantification of proinflammatory cytokines IL-6 and TNF-α in mPFC lysates, showing that SC79 reinstates the neuroinflammatory milieu suppressed by DLT. Data are presented as mean ± SEM (n = 7–10 mice per group for behavior; n = 3 independent biological pools; each pool contains tissue from 3–4 mice). Statistical significance was assessed using one-way ANOVA, followed by Tukey’s post-hoc test or two-tailed Student's t-test, where appropriate. *P < 0.05, **P < 0.01, ***P < 0.001, ****P < 0.0001. ns, not significant. For behavioral and molecular reversal experiments, statistical comparisons were performed between the SCZ + DLT and SCZ + DLT + SC79 groups. For the behavioral and molecular reversal experiments, statistical comparisons were performed between the SCZ + DLT and SCZ + DLT + SC79 groups. For the MWM reversal experiment, SC79 was administered 30 min before each acquisition session and before the probe trial

To determine whether AKT inhibition is essential for the therapeutic action of DLT, we conducted a pharmacological reversal experiment using SC79, a specific AKT activator. Western blot analysis confirmed the in vivo efficacy of SC79, demonstrating dose-dependent increases in AKT phosphorylation following intra-mPFC infusion, with 100 μM achieving robust activation (Fig. 7A–C). We then assessed whether AKT reactivation could negate the therapeutic effects of DLT in SCZ mice. Notably, coadministration of SC79 and DLT fully abrogated DLT’s therapeutic benefits across all behavioral domains. While DLT alone rescued social interaction deficits, SC79 co-treatment reinstated these impairments (Fig. 7D). Similarly, SC79 reversed DLT-mediated improvements in hyperactivity (Fig. 7E) and cognitive function in the MWM test (Fig. 7F, G). This behavioral deterioration was paralleled at the molecular level: SC79 nullified DLT’s neuroprotective effects, promoting a proapoptotic profile (increased Bax, decreased Bcl-2) and reducing levels of BDNF and TH (Fig. 7H, I). Furthermore, SC79 reinstated the neuroinflammatory state by increasing IL-6 and TNF-α expression, previously suppressed by DLT (Fig. 7J). These results provide compelling evidence that suppression of AKT signaling is critical for the therapeutic efficacy of DLT; artificial reactivation of this pathway is sufficient to render DLT ineffective at both behavioral and molecular levels.

## Discussion

In this study, we established that the widely used antihistamine DLT robustly ameliorated a comprehensive spectrum of SCZ-like behaviors in a preclinical model. We provided a novel and detailed mechanism for this therapeutic action, demonstrating that DLT functions by interrupting a pathogenic signaling cascade initiated by the 5-HT2AR within the mPFC. Given that mPFC LTP is essential for ​working memory consolidation​ and ​cognitive flexibility [24–26], our data demonstrate that DLT rectifies core cognitive endophenotypes of schizophrenia by specifically targeting neuron-specific 5-HT2AR dysregulation. ​Mechanistically, our data converged to show that DLT’s efficacy is mediated through suppression of the PI3K/AKT/mTOR pathway, which in turn normalizes 5-HT2AR expression and reverses downstream neuroinflammation, apoptosis, and synaptic plasticity deficits.

A pivotal discovery of our study was the specific upregulation of 5-HT2AR within the mPFC neuronal population in our SCZ model. The literature on 5-HT2AR expression in SCZ postmortem brains is inconsistent, with conflicting reports of increased, decreased, or unchanged receptor density [27–31]. The conflicting reports regarding 5-HT2AR density in postmortem SCZ brains are likely confounded by several clinical variables, including disease chronicity, lifetime exposure to antipsychotics (many of which chronically downregulate 5-HT2A receptors), and the inherent 'dilution effect' of analyzing bulk tissue homogenates. By utilizing single-nucleus resolution, our study circumvents this bulk-tissue limitation, allowing us to pinpoint the highly specific pathological upregulation of 5-HT2AR within vulnerable neuronal subpopulations. However, more recently, highly selective 5-HT2AR antagonists have been evaluated in the treatment of a wide range of other psychiatric disorders [32–35]. Our findings, enabled by the resolution of snRNA-seq, offer a compelling explanation for this variance: the salient pathology may not be a global change in receptor density, but rather a precise dysregulation within a specific cellular compartment—neurons. The concurrent upregulation of 5-HT2AR in both excitatory pyramidal neurons and inhibitory interneurons implies a complex, circuit-level disruption in SCZ. Pathological 5-HT2AR hyperactivation on pyramidal cells typically drives excessive cortical excitability. Simultaneously, aberrant signaling in local inhibitory interneurons may disrupt feed-forward or feed-back inhibition loops, collectively culminating in the profound excitation/inhibition (E/I) imbalance characteristic of the SCZ cortex. This neuron-centric pathology is further supported by our immunofluorescence data and is directly linked to functional consequences, as evidenced by the concurrent synaptic damage and ultrastructural deficits that DLT were able to rescue. Importantly, our neuron-specific finding provides a mechanistic rationale for the clinical exploration of highly selective 5-HT₂AR antagonists​ (e.g., pimavanserin, MIN-101) ​in neuropsychiatric disorders​ [35, 36]. The localization of pathological 5-HT₂AR overexpression to mPFC neurons—key regulators of cognitive-affective processing—suggests that cell-type-precise modulation may overcome historical limitations of non-selective agents.

Critically, our study advances beyond this correlation to establish a causal role for neuronal 5-HT2AR in driving SCZ-like behaviors. Through AAV-mediated overexpression, we demonstrated that elevating 5-HT2AR levels exclusively in mPFC neurons was sufficient to recapitulate the entire behavioral phenotype of the pharmacological SCZ model. While we acknowledge that viral overexpression does not perfectly mimic the complex neurodevelopmental pathology of SCZ, the recapitulation of LTP deficits suggests that 5-HT2AR upregulation is a sufficient driver of this specific synaptic impairment. This gain-of-function experiment provides strong evidence that 5-HT2AR dysregulation in this circuit is a key contributor of disease pathology and not merely an epiphenomenon. The ability of DLT to rescue these genetically induced deficits powerfully anchors its mechanism of action to direct the counter-regulation of the 5-HT2AR-driven cascade.

Our research identified a potential signaling axis between 5-HT2AR and the PI3K/AKT/mTOR signaling pathway in the context of SCZ. Although this pathway is a well-known regulator of cell survival and plasticity [37–39], its direct link with 5-HT2AR in SCZ pathology remains elusive. Our integrated transcriptomic and biochemical evidence demonstrated that 5-HT2AR activation potently engages this pathway. Moreover, the observation that DLT reduces not only the phosphorylation of key pathway nodes, such as AKT and mTOR, but also the expression of 5-HT2AR suggests the existence of a pathogenic feed-forward loop. In this model, the receptor and its downstream pathway may mutually sustain a state of hyperactivity, a vicious cycle in which the DLT effectively breaks.

Engagement of the PI3K/AKT/mTOR pathway provides a coherent molecular narrative of the downstream pathological outcomes. We showed that the activation of this axis culminates in a proinflammatory cellular environment, a proapoptotic state, as evidenced by the Bax/Bcl-2 ratio, and, most functionally relevant, a severe impairment in LTP, a key cellular mechanism for learning and memory [40–43]. This establishes a seamless logical progression from receptor dysregulation to synaptic failure. DLT’s capacity to intercept this cascade at every critical juncture underscores their therapeutic potential. Importantly, beneficial behavioral and molecular effects of DLT were still detectable during the post-treatment testing window. However, because we did not perform a dedicated pharmacokinetic or washout study, we cannot distinguish persistent circuit-level remodeling from residual pharmacodynamic effects. Future studies should address the durability of these effects more directly.

The evidence of the proposed mechanism was provided by pharmacological reversal experiments. By employing the AKT activator SC79 [44, 45], we successfully abolished the therapeutic benefits of DLT. This occlusion experiment demonstrated that the behavioral efficacy of DLT is critically dependent on its ability to inhibit the AKT pathway; when AKT signaling is artificially restored, DLT is rendered ineffective. These data support the PI3K/AKT/mTOR pathway as a key mediator of DLT’s therapeutic effects in our experimental context.

The findings of this study have significant translational implications. We provided a preclinical rationale for repurposing desloratadine, a drug with an exemplary safety profile for the treatment of SCZ, as an adjunctive therapy for refractory negative and cognitive symptoms. Furthermore, this study supports the 5-HT2AR-PI3K/AKT/mTOR axis as a potentially druggable target. However, this study has some limitations. A limitation is that snRNA-seq libraries were generated from pooled animals, precluding animal-level pseudo-bulk inference; we therefore treat the snRNA-seq results as exploratory and support key conclusions with independent animal-level validations. The MK-801 model does not capture the full neurodevelopmental complexity of human SCZ. Additionally, this study was conducted exclusively in male mice; given the known sexual dimorphism in SCZ, future studies must validate these findings in females. Although our updated snRNA-seq analysis successfully sub-clustered excitatory and inhibitory neuronal populations, future in vivo investigations utilizing specific Cre-driver lines (e.g., PV-Cre, SST-Cre) are required to further dissect the precise contribution of discrete interneuron subtypes. Regarding the mechanism, while our pharmacological reversal with SC79 supports the necessity of AKT signaling, we acknowledge that without genetic loss-of-function (e.g., shRNA) studies, the link between 5-HT2AR and PI3K/AKT remains correlative. It is important to acknowledge that DLT is a potent H1 receptor antagonist. Although our snRNA-seq dataset highlighted the specific upregulation of 5-HT2AR, highly sensitive transcriptomic and spatial datasets, such as those from the Allen Brain Atlas [46], confirm the expression of Hrh1 in the mPFC. Therefore, we cannot exclude the possibility that H1 receptor blockade contributes to the observed behavioral rescue. Desloratadine’s therapeutic effects in this model may result from a synergistic action: counteracting the pathologically upregulated 5-HT2AR signaling while simultaneously exerting anti-inflammatory or neuromodulatory effects via constitutive H1R blockade.

In conclusion, this study demonstrated that desloratadine rescued SCZ-like phenotypes by inhibiting the pathogenic 5-HT2AR-PI3K/AKT/mTOR signaling axis in the mPFC. We provide causal evidence that this axis drives SCZ-like behaviors and the underlying cellular pathologies. These findings not only elucidate a powerful neuroprotective mechanism for a repurposed drug, but also highlight a pivotal feedback loop that represents a promising new therapeutic target for schizophrenia. Future studies should aim to validate these mechanisms in human-derived cellular models and explore the clinical potential of DLT for treating the multifaceted symptoms of schizophrenia.

## Supplementary Information

Below is the link to the electronic supplementary material.

Supplementary file1 (DOCX 10391 KB)

## Data Availability

Data available on request from the corresponding author upon reasonable request.
