# Supplementary material for: Desloratadine Rescues Schizophrenia-like Phenotypes by Inhibiting the Pathogenic 5-HT2AR-PI3K/AKT/mTOR Signaling Axis
Source: Mol Neurobiol. 2026 Jun 24;63(1):716. doi: 10.1007/s12035-026-06027-z (PMC13294246; doi:10.1007/s12035-026-06027-z)
Supplement: Supplementary file 1 — Supplementary file1 (DOCX 10391 KB) [file 12035_2026_6027_MOESM1_ESM.docx]

**Fig. S
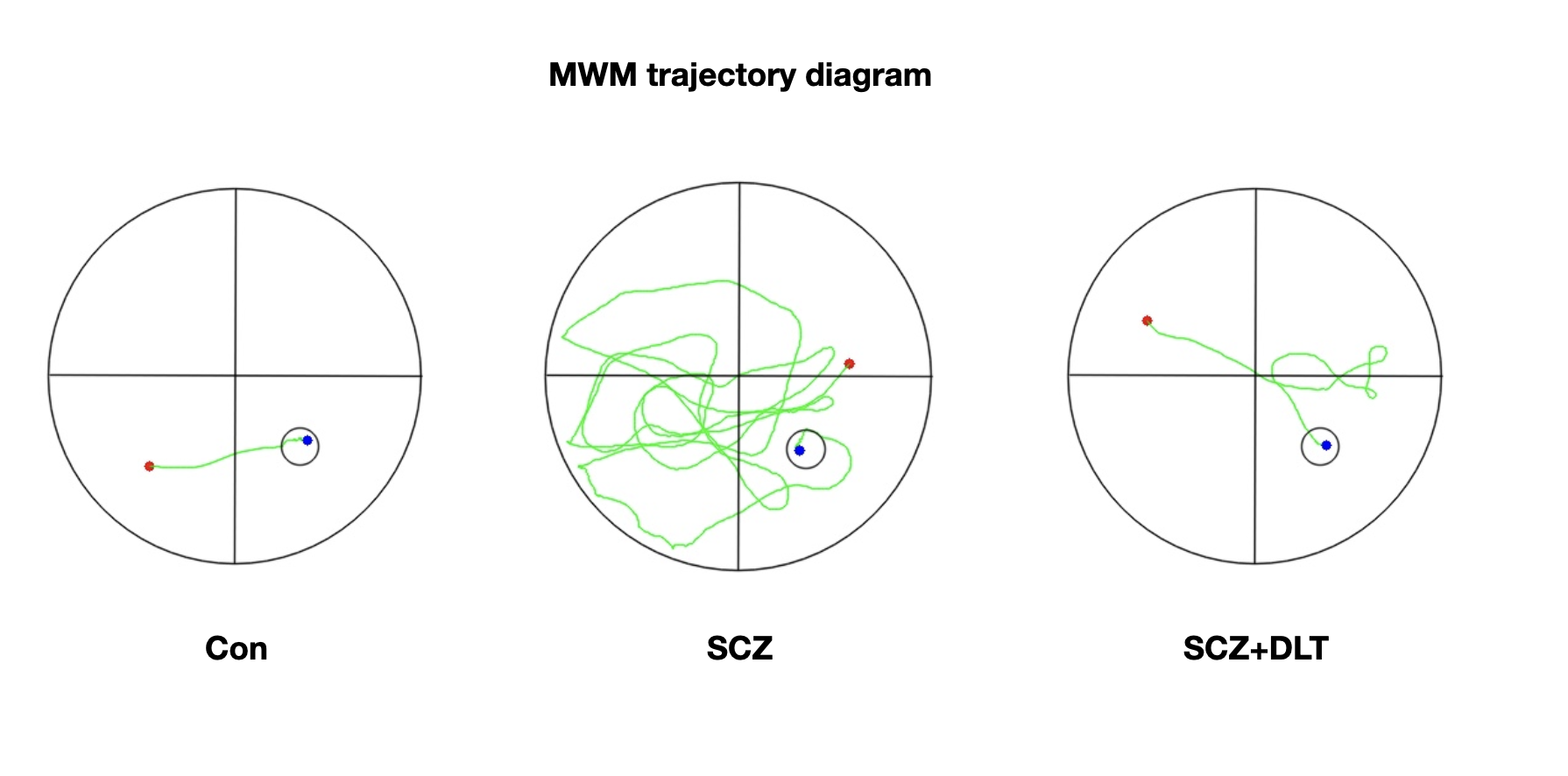
1. Representative swim trajectories in the Morris water maze.** Representative swim paths during the probe trial are shown for the Con, SCZ, and SCZ+DLT groups. The green line indicates the swim trajectory; the blue marker indicates the former platform location/target; and the red marker indicates the start position. These trajectories illustrate more direct target-oriented searching in Con mice, disorganized searching in SCZ mice, and improved search patterns in SCZ+DLT mice.

**
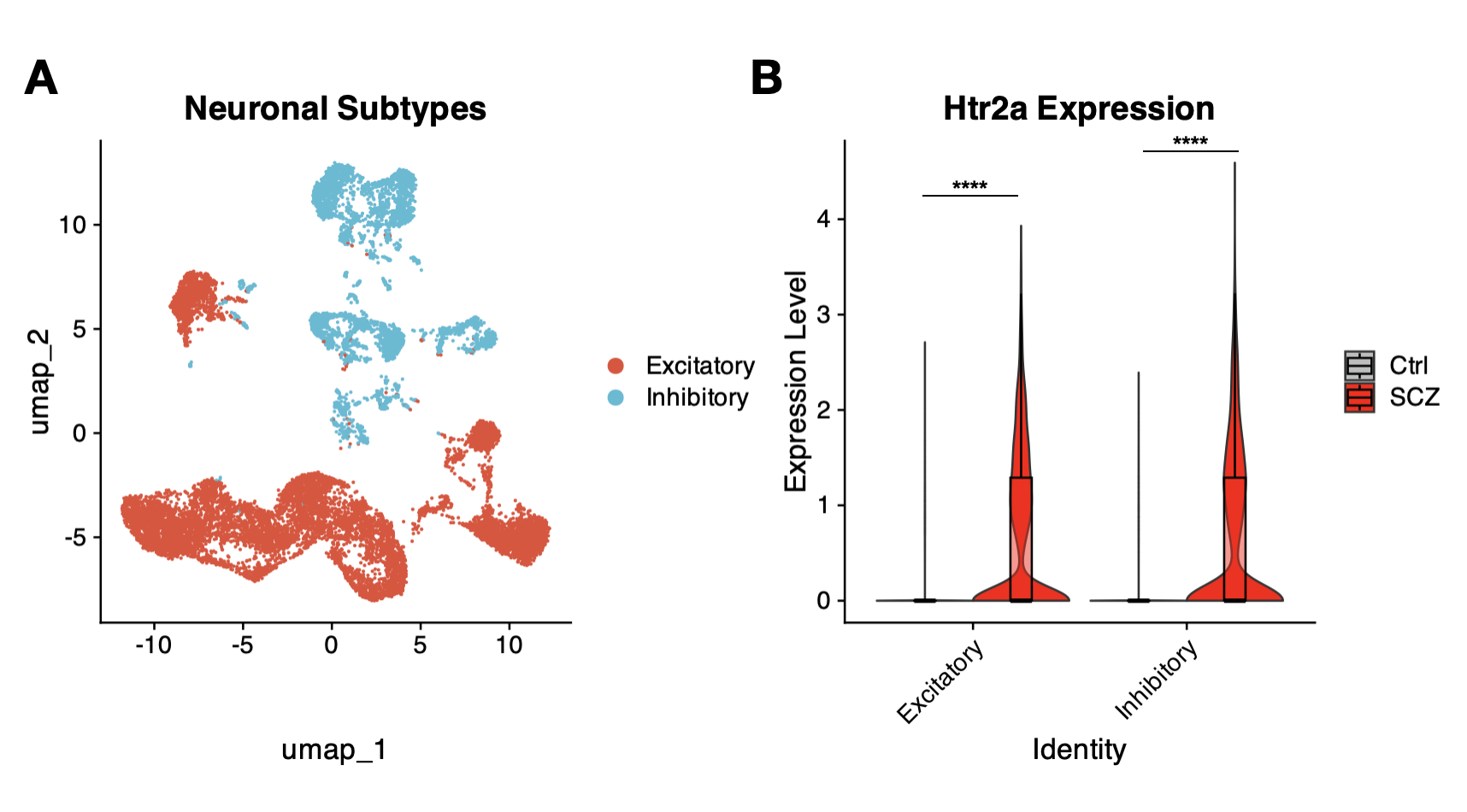
**

**Fig. S2. Sub-clustering analysis identifies *Htr2a* upregulation in both excitatory and inhibitory neurons as sources of *Htr2a* upregulation in the SCZ model. (A)** UMAP visualization of mPFC neurons re-clustered into two major subtypes: Excitatory neurons (*Slc17a7*+, red) and Inhibitory neurons (*Gad1*/*Gad2*+, blue). **(B)** Violin plots showing the expression levels of *Htr2a* in Excitatory and Inhibitory neurons, split by experimental group (Grey: Ctrl; Red: SCZ). Note that the pathogenic upregulation of *Htr2a* is highly significant in both the Excitatory neuron population (avg_log2FC = 1.86, *****p* < 0.0001) and the Inhibitory neuron population (avg_log2FC = 2.25, *****p* < 0.0001, Wilcoxon rank-sum test).

**Fig. S3. Co-expression of *Htr2a* and PI3K/AKT/mTOR pathway activity.** Dot plot showing the expression of *Htr2a* and the PI3K/AKT/mTOR signaling module score across neuronal subtypes. Note that both *Htr2a* and Pathway Activity are concurrently upregulated (red/large dots) specifically in the **SCZ-Excitatory** population, supporting a functional link.


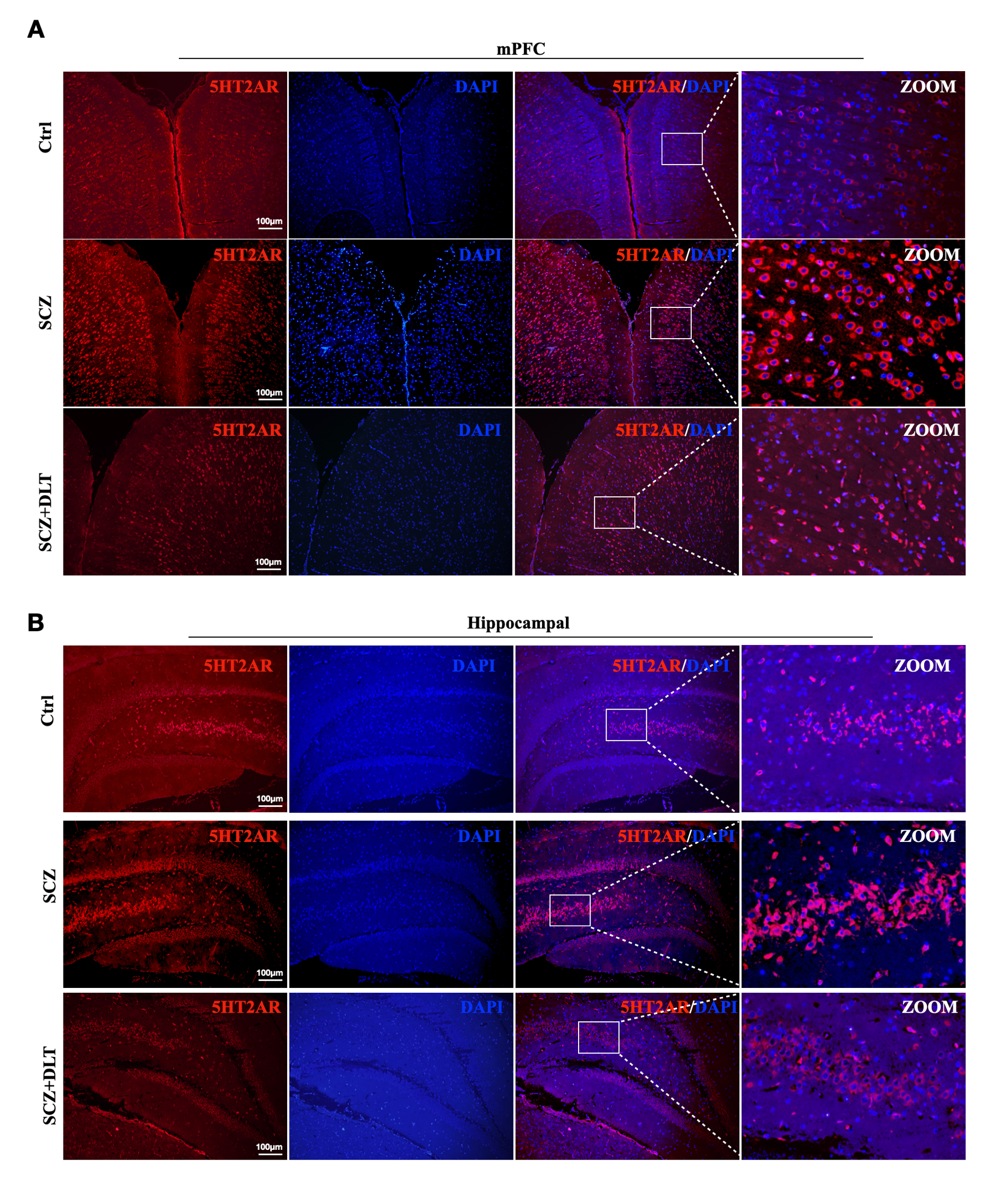


**Fig. S4. Immunofluorescence staining of 5-HT2AR across experimental groups.** Representative confocal images showing 5-HT2AR (red) and DAPI (blue) staining in the **mPFC (A)** and **Hippocampus (B)** from Control, SCZ, and SCZ+DLT mice. **(A)** In the mPFC, 5-HT2AR expression is visibly increased in the SCZ group compared to Controls and is downregulated following DLT treatment. **(B)** In the Hippocampus, a similar trend of upregulation in SCZ and downregulation by DLT is observed. Scale bars = 100 μm (low magnification).


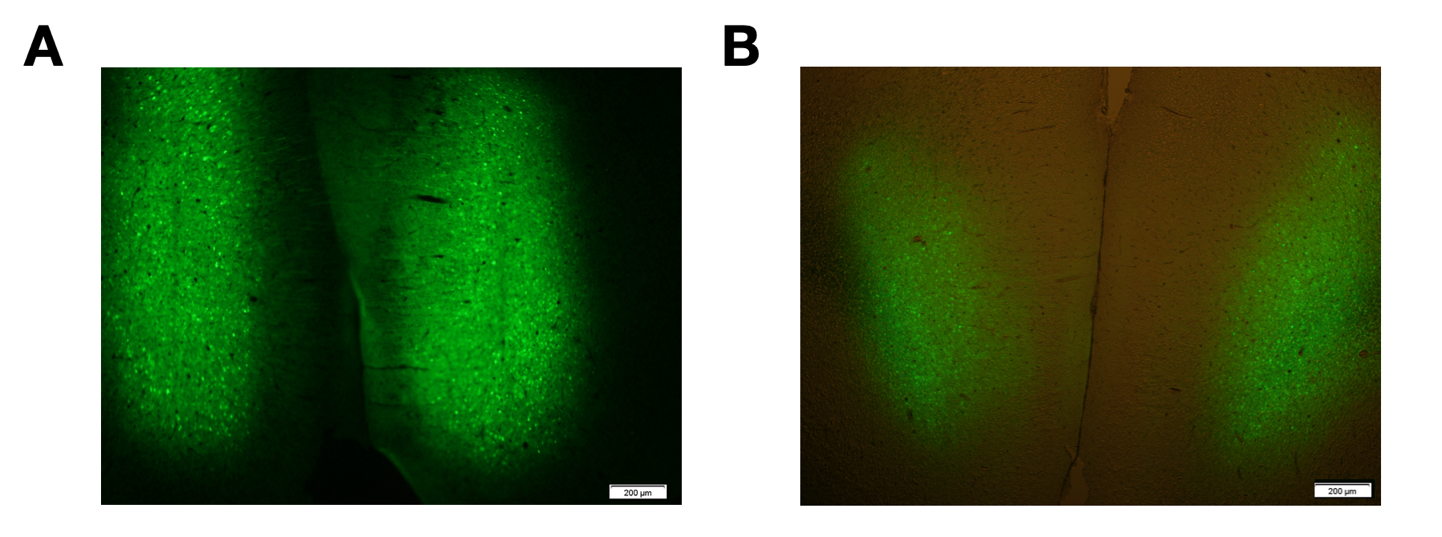


**Fig. S5. Verification of AAV injection site in the mPFC.** **(A)** Coronal schematic from the mouse brain atlas (approx. Bregma +1.9 mm) indicating the target medial prefrontal cortex (mPFC) region. The red dashed box represents the approximate field of view captured in the fluorescence images. **(B)** Representative GFP fluorescence image showing accurate bilateral AAV expression spanning the prelimbic (PL) and infralimbic (IL) cortices, aligned symmetrically across the anatomical midline.

**
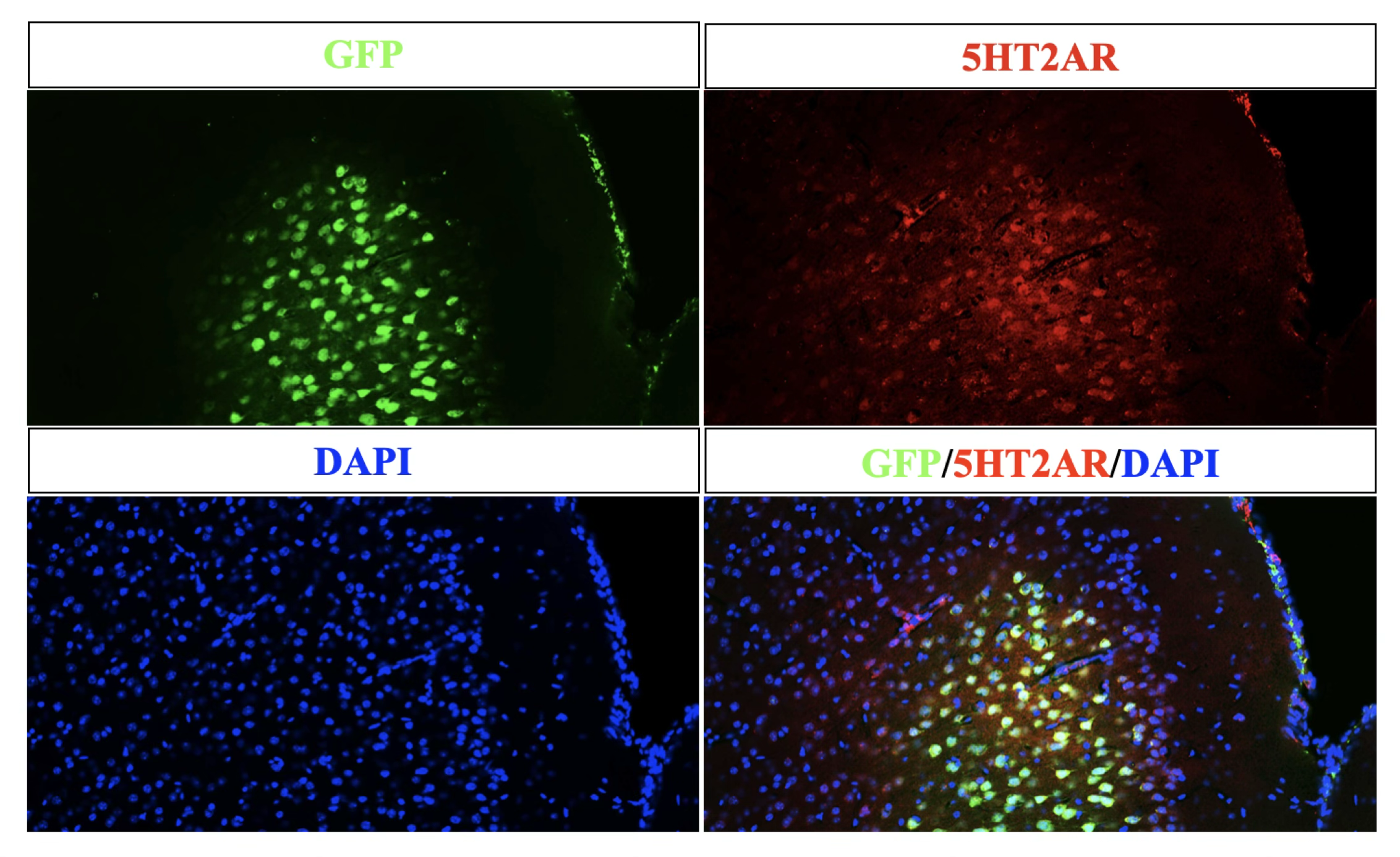
**

**Fig. S6. In situ validation of 5-HT2AR antibody specificity using AAV-mediated overexpression.** Representative immunofluorescence images of the mPFC from an AAV-hSyn-5HT2AR-EGFP injected mouse. The virally infected region is indicated by native GFP expression (green). Staining with the 5-HT2AR antibody (red) reveals a massive, spatially restricted amplification of signal that co-localizes with the GFP-positive infected area, compared to the baseline physiological staining in adjacent non-injected cortical regions. This robust overlap confirms the target specificity of the antibody in fixed tissue preparations.
